# Integration of Ki-67 index into AJCC 2018 staging provides additional prognostic information in breast tumours candidate for genomic profiling

**DOI:** 10.1038/s41416-019-0656-6

**Published:** 2019-11-29

**Authors:** Elena Vissio, Jasna Metovic, Simona Osella-Abate, Luca Bertero, Giuseppe Migliaretti, Fulvio Borella, Chiara Benedetto, Anna Sapino, Paola Cassoni, Isabella Castellano

**Affiliations:** 1 Department of Medical Sciences, University of Turin, Via Santena 7, 10126 Turin, Italy; 2 Department of Oncology, Pathology Unit, University of Turin, Via Santena 7, 10126 Turin, Italy; 3 Department of Public Health and Pediatric Sciences, School of Medicine, University of Turin, 10126 Turin, Italy; 4 Department of Surgical Sciences, Gynecology and Obstetrics 1, AOU Città della Salute, 10126 Turin, Italy; 5 Pathology Division, Candiolo Cancer Institute, FPO-IRCCS, Str. Prov. 142, 10060 Candiolo, Italy

**Keywords:** Oncology, Medical research

## Abstract

**Background:**

The Eighth edition of the American Joint Committee on Cancer (AJCC) staging system (2018) for breast cancer (BC) introduced the prognostic stage. Moreover, multigene assessment has been indicated to tailor staging in T1/T2/N0, ER-positive/HER2-negative BC. However, many National Health Systems do not provide reimbursement for routine testing. The aim of this study was to assess whether Ki67 proliferation index is prognostically relevant for patients’ candidacy for molecular testing.

**Methods:**

A retrospective series of 686 ER+/HER2− BC were reclassified using AJCC 2018, and in the group of 521 patients for which AJCC 2018 recommends molecular evaluation, we assessed the prognostic efficacy of a prognostic stage enriched by Ki67 (Ki67-PS), considering Ki67 <20% an alternative to recurrence score <11 provided by Oncotype DX.

**Results:**

We found that a group of BCs (35.6%, 58/163) assigned to IB stage by prognostic score were down classified to IA with Ki67-PS. The outcome of these 58 cases overlapped with that of lesions classified as stage IA using prognostic stage, showing a significantly better prognosis compared to IB tumours (HR = 2.79, *p* = 0.003).

**Conclusions:**

These data suggest that Ki67 may be a reliable marker to enrich the 2018 AJCC prognostic score in BC patients’ candidacy for genomic profiling.

## Background

Breast cancer (BC) is the most common cancer in women. The clinical approach to this disease has varied over the years from radical surgery and aggressive oncological therapy, to the minimal patient-tailored effective treatment.^1,2^

Recently, several studies demonstrated that the biological phenotype of the tumour may be a superior prognostic variable than lymph node staging.^3^ In particular, Mittendorf et al. described that, among T1 BC patients, oestrogen receptor (ER) status and histological grade are better predictors of survival than the presence of small-volume nodal metastases.

Accordingly, the Eighth edition of the American Joint Committee on Cancer (AJCC) staging system, published in 2018, proposed the use of a dual approach based on the traditional anatomic stage (AS) (i.e. tumour size, lymph node status), which remains unchanged from the Seventh AJCC edition and the novel prognostic stage (PS). This latter takes into account biological information, such as ER, progesterone receptor (PR), HER2 status and histological grade and integrates them with AS.

To optimise patient care and in particular to allow appropriate treatment de-escalation, AJCC 2018 recommends molecular profiling in T1/T2 tumours without lymph node metastases and ER-positive/HER2-negative status. Specifically, four tools have been recommended: Oncotype DX® (level of evidence, I), Mammaprint®, Endopredict®, and Breast Cancer Index® (level of evidence, II). In particular, the AJCC suggested that independently from AS, ER-positive/HER2-negative tumour should be reclassified as stage IA in case of recurrence score (RS) <11 by Oncotype DX®.

To date, in many European countries, including Italy, none of these molecular tests is reimbursed by the National Health System hampering the prompt translation of AJCC 2018 recommendations into the routine clinical practice. In addition, even if approved, these tests could burden the budget sustainability of pathology laboratories.

The proliferation index, assessed using Ki67, is considered an important prognostic biomarker in BC.^4^ Ki67 is typically useful in ER-positive/HER2-negative BC to discriminate, together with PR, luminal A from luminal B cases, as recommended by St. Gallen guidelines.^5^ Determination of Ki67 by immunohistochemistry (IHC) is routinely used to integrate the histology report and to add prognostic information, despite some criticism regarding its reproducibility^6^ and different cut-off values proposed in literature.^5,7,8^

Since most of the genes assessed by the previously listed molecular assays are related to cell proliferation, we hypothesised that a proliferative marker like Ki67 could partly substitute information obtained by genomic profiling.

The aim of the present study was to evaluate the efficacy of a Ki67-integrated AJCC 2018 PS (Ki67-PS) for prognostic assessment of patients’ candidacy for molecular assays. In particular, we first reclassified a retrospective series of ER+/HER2− BC using both AJCC AS and PS. Then, in the subgroup of patients’ candidacy for multigene panel evaluation according to AJCC, we tested the prognostic efficacy and reliability of Ki67-PS.

## Methods

### Case series

We retrospectively evaluated 686 ER+/HER2− BC patients who underwent conservative surgery at the Breast Unit of “Città della Salute e della Scienza” University Hospital (Turin, Italy) from April 1998 to December 2012. Data concerning tumour diameter, lymph node involvement, tumour grade, histological type, ER, PR, HER2 and Ki67 expression levels were obtained from the pathological reports. In addition, type of therapy and follow-up status were collected from clinical reports. All the cases were anonymously recorded into a dedicated database, and data were accessed anonymously. The study was conducted in accordance with The Code of Ethics of the World Medical Association (Declaration of Helsinki) and within the guidelines and regulations defined by the Research Ethics Committee for human Biospecimen Utilization (Department of Medical Sciences—ChBU) of the University of Turin. Considering the retrospective nature of this research protocol, which involved only already existing medical data that were previously anonymised with no impact on patient care, no specific written informed consent was required by the Committee.

### Immunohistochemistry

Tissue sections were routinely immunostained using an automated slide processing platform (Ventana BenchMark AutoStainer, Ventana Medical Systems, Tucson, AZ, USA) with the following primary antibodies: prediluted anti-ER rabbit monoclonal antibody (SP1, Ventana Medical Systems), prediluted anti-PgR rabbit monoclonal antibody (1E2, Ventana Medical Systems), and anti-Ki67 mouse monoclonal antibody (MIB1, diluted 1:50, Dako). Evaluation of HER2 expression was performed by an anti-HER2 polyclonal antibody (A0485, diluted 1:800, Dako). Fluorescence in situ hybridisation was performed to define HER2 status in IHC equivocal cases (score 2+).^9^ Positive and negative controls were included for each IHC run.

### Pathological evaluation

Tumour size was dichotomised at 15 mm, as suggested by previous studies.^10,11^

Cut-off for ER and PR positivity was determined at <1%, according to the Consensus of St. Gallen 2011.^12^ HER2 was evaluated as recommended by the American Society of Clinical Oncology/College of American Pathologists.^13^ Ki67 proliferation index was assessed on surgical specimens and a minimum of 1000 cells were evaluated.^4^ The surrogate of molecular subtypes obtained from ER, PR and HER2 IHC expression is summarised in Supplementary Table 1. Luminal subtypes were defined according to St. Gallen proposal^5^ using a Ki67 cut-off value of 20% in line with previously published studies.^7,14^

### Anatomic and prognostic staging

All cases (*n* = 686) were first staged using AS and PS, then BC in which further molecular testing (T1/T2, N0, M0) would be recommended according to AJCC 2018 were selected (*n* = 521).^15^ We hypothesised that the expression of Ki67 may provide prognostic information related to those obtained by Oncotype DX. Thus, in analogy to Oncotype DX® RS <11, we selected a value of Ki67 <20% to identify tumours staged IIA and IB, which could be reclassified as IA. In case of Ki67 values ≥20%, as for RS ≥ 11 the PS was not modified.

### Statistical analysis

Categorical data were described as counts and percentages. Disease-free interval (DFI) was determined from the date of diagnosis to the date of first recurrence (either locoregional recurrence or distant metastasis), or if no recurrence occurred, analysis was censored at the time of last follow-up. DFI was estimated with the Kaplan–Meier analysis. The Cox model was used to assess the prognostic value of a series of patient and tumour characteristics. Hazard ratios (HRs) and 95% confidence intervals (CIs) were also calculated. The proportional hazard assumption (Schoenfeld residuals) was always satisfied. The performance of the AJCC 2018 was informally compared through the Harrell *c* or the Somer *D* discrimination statistics in which the higher value was representative of better system performance. The Akaike information criterion was also computed, a lower value indicating the better performance of the model. Data were analysed with Stata (version 15; Stata Corporation, College Station, TX, US). Agreement among different classification systems were performed using Cohen *K*. A two-sided *P* value of <0.05 was considered statistically significant. All statistical tests were two sided.

## Results

### Clinicopathological characteristics

Clinical and pathological information of 686 patients are reported in Supplementary Table 2. Briefly, 59.5% of the tumours had a diameter <15 mm and 85% were classified as pT1; of these, 42.1% were well differentiated (G1) and 11.4% were poorly differentiated (G3). Lymph nodes resulted free of metastases in 76.1% of patients. The proliferation rate was low (Ki67 < 20%) in 74.1% of cases. Most of tumours expressed PR and 59.3% were classified as Luminal A. All patients were treated by conservative surgery followed by radiotherapy. Hormonal therapy was administrated to 95.2% of patients, while 23% received chemotherapy. Distant or local relapse was observed in 58 patients (8.4%) and 21 died of BC (3.1%).

### Classification using AJCC 2018

**Fig. 1:**
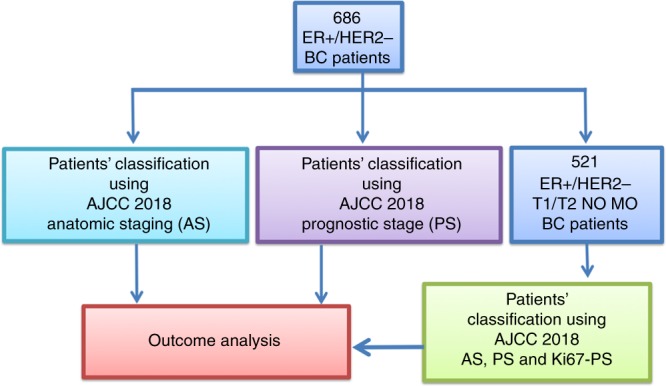
Study flowchart.

Patients were staged according to the AJCC 2018 anatomic staging (Fig. 1—AS). According to this system, 468 (68.2%), 28 (4.1%), 132 (19.2%) and 39 (5.7%) of tumours were staged as IA, IB, IIA and IIB, respectively, whereas 19 (2.7%) were in stage III (Supplementary Table 3).

Then we re-staged the tumours using AJCC 2018 PS (Fig. 1—PS). Applying this staging system, the majority of tumours were still classified as IA (63.7%); however, the PS reassigned to IA and IB stage the majority of patients previously classified as IB or IIA by AS (Supplementary Table 3).

Conversely, 57 cases changed from IA by AS to IB (51) and IIA (6) according to PS. Only 15 out of 39 cases staged as IIB by AS were confirmed by PS, while 14 cases were upstaged into IIIA, 2 were assigned to IIIB and 8 were down staged to IB (Supplementary Table 3).

Supplementary Table 4 summarised the results obtained by AS and PS, grouping stage I–II–III patients. Using the new prognostic classification proposed by AJCC, the majority of patients of our series were shifted in stage I [*K* = 0.38, 95% CI (0.33–0.41)]. In particular, using the AS 5.6% of cases were stage IB, the rate increased to 27.2% using the PS.

### Ki67-integrated PS

We selected 521 patients with BC staged as T1/T2N0M0 who were potential candidates for molecular assessment following AJCC 2018. Differences between AJCC 2018 AS and PS are summarised in Supplementary Table 5. In this subgroup, Ki67 proliferation index was used to integrate the PS with additional information regarding biological aggressiveness (Ki67-PS) (Fig. 1—Ki67-PS).

Clinical and pathological information of this patient group are reported in Table 1. As shown in Table 2, 411 patients remained assigned to IA stage using both PS and Ki67-PS, while 58 out of 89 (65.2%) and 3 out of 19 (15.8%) BCs previously classified as IB and IIA, respectively, were down staged to IA, using Ki67-PS. In terms of absolute differences, 61/521 (approximately 12%) patients were differently classified.

**Table 1:** Clinical and pathological characteristics of patients candidate for molecular profiling.

	No. of patients 521	%
*Diameter*		
<15 mm	343	65.8
≥15 mm	178	34.2
*pT*		
1	468	89.8
2	53	10.2
*Grade*		
1	231	44.3
2	244	46.8
3	46	8.8
*Ki67*		
<20%	404	77.5
≥20%	117	22.5
*PR*		
Negative	33	6.3
Positive	488	93.7
*Subtype*		
Luminal A	319	61.2
Luminal B	202	38.8
*Chemotherapy*		
No	468	89.8
Yes	53	10.2
*Recurrences*		
No	491	94.2
Yes	30	5.8

*PR* progesterone receptor

**Table 2:** Classification of 521 BC patients according to prognostic stage Eighth edition AJCC 2018 and prognostic stage modified using Ki67 (Ki67-PS).

AJCC 2018 prognostic stage	AJCC 2018 prognostic stage modified by Ki67 (Ki67-PS)					
	IA	IB	IIA	IIB	IIIA	Total
IA	411	0	0	0	0	411
IB	**58**	31	0	0	0	89
IIA	**3**	0	16	0	0	19
IIB	0	0	0	0	0	0
IIIA	0	0	0	0	2	2
IIIB	0	0	0	0	0	0
IIIC	0	0	0	0	0	0
Total	472	31	16	0	2	521

Bold values indicate the number of patients that were differently classified

Table 3 summarises the results obtained by the three different staging systems, grouping stage I–II–III patients. Prognostic staging (95.9%) and Ki67-PS (96.5%) moved to stage I the majority of BCs. In general, we observed an overlap between PS and Ki67-PS, although stage IA counted more cases (411 vs 472) according to Ki67-PS.

**Table 3:** Classification of 521 BC patients following Eighth edition AJCC 2018 (AS, PS and Ki67-PS).

	Stage I		Stage II		Stage III
AJCC 2018 anatomic stage	468		53		0
	IA	IB	IIA	IIB	IIIA
	468	0	53	0	0
AJCC 2018 prognostic stage	Stage I		Stage II		Stage III
	500		19		2
	IA	IB	IIA	IIB	IIIA
	411	89	19	0	2
AJCC 2018 prognostic stage with Ki67	Stage I		Stage II		Stage III
	503		16		2
	IA	IB	IIA	IIB	IIIA
	472	31	16	0	2

### Outcome analysis according to different staging systems

**Fig. 2:**
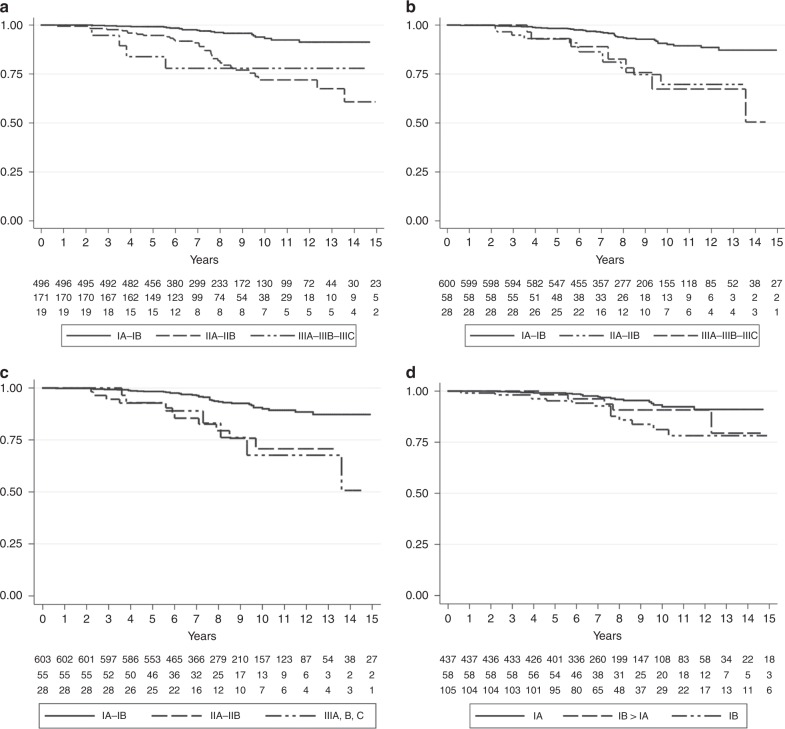
Disease-free interval (DFI) of stage I–II–III assessed using AJCC 2018 anatomical stage (log-rank test *p* < 0.001) (**a**), prognostic stage (log-rank test *p* < 0.001) (**b**) and Ki67-PS (log-rank test *p* < 0.001) (**c**) (Kaplan–Meier analysis). DFI of stage IA and IB assessed using prognostic stage and of stage IA obtained from downstaging of IB using Ki67-integrated prognostic score (Ki67-PS) (log-rank test *p* < 0.001) (Kaplan–Meier analysis) (**d**).

To understand which staging system could be more accurate to predict the prognosis in ER+ BC patients, we used Kaplan–Meier analysis (Fig. 2a–c). Only PS and Ki67-PS clearly distinguished stage I from stage II and III (log-rank test *p* < 0.001) (Fig. 2b, c, respectively). In addition, a significant difference of DFI among stages (I–II–III) was observed at univariate analyses regardless of the staging system used (Table 4).

**Table 4:** Univariate analyses on DFI across different staging systems proposed by Eighth edition AJCC 2018 and using Ki67-integrated PS.

System classification		HR	CI	*p* value
AJCC 2018 anatomic stage (AS) Harrell *c* test 0.6993; AIC 672.6299	I	1		
	II	4.54	2.63–7.82	<0.001
	III	4.62	1.58–13.48	0.005
AJCC 2018 Prognostic stage (PS) Harrell *c* test 0.6993; AIC 672.6299	I	1		
	II	3.44	1.80–6.57	<0.001
	III	3.87	1.73–8.66	0.005
AJCC 2018 PS integrated by Ki67 (Ki67-PS) Harrell *c* test 0.6094; AIC 674.1635	I	1		
	II	3.27	1.67–6.36	0.001
	III	3.79	1.70–8.47	0.001
AJCC 2018 PS and Ki67-PS Harrell *c* test 0.6265	IA	1		
	IB > IA	1.66	0.62–4.44	0.307
	IB	2.79	1.41–5.53	0.003

Based on PS, DFI was significantly different in stage IA and IB (log-rank test *p* < 0.001) (Fig. 2d). In particular, the 58 cases that were down staged from IB to IA using Ki67-PS showed a favourable outcome, similar to those classified as stage IA (*p* = 0.307) (Fig. 2d, Table 4) and a better prognosis compared to IB lesions (HR = 2.79, *p* = 0.003).

## Discussion

In the present study, a retrospective series of ER+/HER2− BC with long follow-up was reclassified using both Eighth edition AJCC AS and PS. The results obtained confirm that integration of tumour load (size and presence of node involvement) with tumour type (grade and prognostic factors) leads to an increased number of patients classified as stage I, as previously reported.^16,17^ Furthermore, in line with other studies,^18,19^ we found that stage I according to PS clearly identifies a group of patients with a more favourable outcome, distinguishing them from other patients with lesions classified as stage II or III and providing more accurate prognostic information compared with AS.

To further improve patient care and avoid unnecessary treatments, AJCC 2018 recommends the use of multigene profiling in the subset of T1/T2-N0, HER2-negative luminal BCs.

However, in many countries, including Italy, the National Health System does not reimburse these tests, hampering the prompt translation of AJCC 2018 recommendations into the routine clinical practice.

In the absence of molecular assays, Ki67 is to date the only recommended marker, together with PR, that can help oncologists to differentiate luminal A from luminal B surrogate categories.^8^

In the present study, we created a PS integrated with Ki67 (Ki67-PS), hypothesising that expression of Ki67 may stratify patients similarly to Oncotype DX^®^. Actually, Oncotype DX® is based, among others, on the expression of 5 genes related to proliferation (namely MKI67, STK15, Survivin, CCNB1 and MYBL2), and the association between both RS and single gene expression with the Ki67 IHC levels has previously been addressed.^20–23^

Since use of Oncotype DX® in routine practice requires important financial resources and its cost-effectiveness has been questioned in the literature,^24,25^ especially for low-risk BC patients, Ki67-PS can possibly provide additional information with an inferior burden on National Health System budget.

Several works reported a poor reproducibility of Ki67 assessment due to the use of different clones (e.g. MIB-1, MM1, NCL-Ki-67p)^26^ and different pre-analytic procedures, as well as discordant diagnostic evaluation even in case of dedicated breast pathologists.^27^ To overcome this problem, in Italy, breast pathologists and breast pathological laboratories perform routinely local, regional and national quality controls to standardise pre-analytical and analytical assessment of this marker, according to recommendation by the St Gallen Consensus Conference.^5^ In addition, we and other groups demonstrated that 20% is an optimal cut-off of Ki67 to stratify patients with luminal BCs.^14,28,29^ Thus we hypothesised that tumours showing Ki67 < 20% may be classified as stage IA, similarly to those with RS < 11.

In the present study, we showed that prognostic score clearly separates stage I tumours from the others. However, using the integrated Ki67-PS, 61/521 (12%) patients were down staged from IB (58 patients) and from IIA (3 patients) to IA with an outcome comparable to those classified as stage IA defined by PS in terms of DFI. These data support Ki67 as a possible marker to identify the subgroup of patients with luminal BC with good prognosis in which treatment de-escalation could be considered.

The present study has some limitations that warrant consideration. Its retrospective nature limits the collection of follow-up data. Owing to the small number of patients who died of disease, we could not perform survival analyses. However, to the best of our knowledge, this is the first study that reports effective integration of the newly introduced AJCC 2018 PS system with Ki67 IHC evaluation.

In conclusion, our results confirmed that PS provides better prognostic information compared to AS in luminal BC patients. Moreover, the use of Ki67-integrated PS may be a reliable method to obtain additional prognostic data, enriching the 2018 AJCC system in BC patients’ candidacy for genomic profiling.

## Supplementary information

Supplementary Tables

## Data Availability

The dataset analysed during the current study is available from the corresponding author on reasonable request. Data generated during this study are included in this published article [and its supplementary information files].
