# Supplementary material for: Integration of Ki-67 index into AJCC 2018 staging provides additional prognostic information in breast tumours candidate for genomic profiling
Source: Br J Cancer. 2019 Nov 29;122(3):382–7. doi: 10.1038/s41416-019-0656-6 (PMC7000715; doi:10.1038/s41416-019-0656-6)
Supplement: Supplementary file 1 — Supplementary Tables [file 41416_2019_656_MOESM1_ESM.docx]

**Supplementary Table 1:** Immunohistochemical surrogate breast cancer characterization

| ***BC subtype*** | ***IHC surrogate characterization*** |
| --- | --- |
| Luminal A | ﻿ER and PR positive (>1%)  HER2 negative  Ki-67 <20% |
| ﻿Luminal B (HER2 negative)  Luminal B (HER2 positive) | ﻿ER positive  HER2 negative  and at least one of:  Ki-67 >20%  PR negative  ﻿ER positive  HER2 over-expressed or amplified  Any Ki-67  Any PR |
| HER2 | ﻿HER2 over-expressed or amplified  ER and PR negative |
| Triple negative | ﻿ER and PR negative  HER2 negative |

**Supplementary Table 2:** Clinical and pathological characteristics of all patients

|  | **N. of patients**  **686** | **%** |
| --- | --- | --- |
| **Diameter** |  |  |
| <15 mm | 408 | 59.5 |
| ≥15 mm | 278 | 40.5 |
| **pT** |  |  |
| 1 | 583 | 85.0 |
| 2 | 102 | 14.9 |
| 3 | 0 | 0 |
| 4 | 1 | 0.1 |
| **pN** |  |  |
| 0 | 522 | 76.1 |
| 1 | 146 | 21.3 |
| 2 | 15 | 2.2 |
| 3 | 3 | 0.4 |
| **Histological Grade** |  |  |
| 1 | 289 | 42.1 |
| 2 | 319 | 46.5 |
| 3 | 78 | 11.4 |
| **Ki67** |  |  |
| <20% | 508 | 74.1 |
| ≥20% | 178 | 25.9 |
| **PR*** |  |  |
| Negative | 45 | 6.6 |
| Positive | 641 | 93.4 |
| **Subtype** |  |  |
| Luminal A | 407 | 59.3 |
| Luminal B | 279 | 40.7 |
| **Hormonal therapy** |  |  |
| No | 33 | 4.8 |
| Yes | 653 | 95.2 |
| **Chemotherapy** |  |  |
| No | 528 | 77 |
| Yes | 158 | 23 |
| **Recurrence** |  |  |
| No  Yes | 628 | 91.6 |
| Yes | 58 | 8.4 |
| **Death** |  |  |
| No | 665 | 96.9 |
| Yes | 21 | 3.1 |

*(PR = Progesterone Receptor)

**Supplementary Table 3:** Classification of 686 BC patients according to Anatomic Stage and Prognostic Stage 8^th^ edition AJCC 2018

|  |  | AJCC 2018 Prognostic Stage | | | | | | |  |
| --- | --- | --- | --- | --- | --- | --- | --- | --- | --- |
|  |  | IA | IB | IIA | IIB | IIIA | IIIB | IIIC | Total |
| AJCC 2018  Anatomic Stage | IA | 411 | 51 | 6 | 0 | 0 | 0 | 0 | 468 (68.2%) |
|  | IB | 26 | 1 | 1 | 0 | 0 | 0 | 0 | 28 (4.1%) |
|  | IIA | 0 | 103 | 23 | 2 | 4 | 0 | 0 | 132 (19.2%) |
|  | IIB | 0 | 8 | 0 | 15 | 14 | 2 | 0 | 39 (5.7%) |
|  | IIIA | 0 | 0 | 4 | 7 | 0 | 2 | 2 | 15 (2.2%) |
|  | IIIB | 0 | 0 | 0 | 0 | 0 | 1 | 0 | 1 (0.1%) |
|  | IIIC | 0 | 0 | 0 | 0 | 1 | 0 | 2 | 3 (0.4%) |
| Total | | 437  (63.7%) | 163  (23.8%) | 34  (4.9%) | 24  (3.5%) | 19 (2.8%) | 5 (0.7%) | 4  (0.6%) | 686 (100%) |

**Supplementary Table 4:** Classification of 686 BC patients following 8^th^ edition AJCC 2018 (AS and PS)

|  | **Stage I** | | **Stage II** | | **Stage III** | | |
| --- | --- | --- | --- | --- | --- | --- | --- |
| AJCC 2018  ANATOMIC STAGE | 496 | | 171 | | 19 | | |
|  | **IA** | **IB** | **IIA** | **IIB** | **IIIA** | **IIIB** | **IIIC** |
|  | 468 | 28 | 132 | 39 | 15 | 1 | 3 |
| AJCC 2018  PROGNOSTIC STAGE | **Stage I** | | **Stage II** | | **Stage III** | | |
|  | 600 | | 58 | | 28 | | |
|  | **IA** | **IB** | **IIA** | **IIB** | **IIIA** | **IIIB** | **IIIC** |
|  | 437 | 163 | 34 | 24 | 19 | 5 | 4 |

K=0.38, IC95% (0.33-0.41)

**Supplementary Table 5.** Classification of 521 BC patients according to Anatomic Stage and Prognostic Stage 8^th^ edition AJCC 2018.

|  |  | AJCC 2018 Prognostic Stage | | | | |  |
| --- | --- | --- | --- | --- | --- | --- | --- |
|  |  | IA | IB | IIA | IIB | IIIA | Total |
| AJCC 2018  Anatomic Stage | IA | 411 | **51** | **6** | 0 | 0 | 468  (89.8%) |
|  | IB | 0 | 0 | 0 | 0 | 0 | 0 |
|  | IIA | 0 | **38** | 13 | 0 | 2 | 53  (10.2%) |
|  | IIB | 0 | 0 | 0 | 0 | 0 | 0 |
|  | IIIA | 0 | 0 | 0 | 0 | 0 | 0 |
|  | IIIB | 0 | 0 | 0 | 0 | 0 | 0 |
|  | IIIC | 0 | 0 | 0 | 0 | 0 | 0 |
| Total | | 411  (78.9%) | 89  (17.1%) | 19  (3.6%) | 0 | 2  (0.4%) | 521  (100%) |
